# Supplementary material for: Endogenous retroelements promote tolerance to dietary antigens
Source: bioRxiv. 2025 Sep 9:2025.09.09.675192. Preprint. [Version 1] doi: 10.1101/2025.09.09.675192 (PMC12439965; doi:10.1101/2025.09.09.675192)
Supplement: 1 [file NIHPP2025.09.09.675192v1-supplement-1.pdf]

bioRxiv preprint doi: https://doi.org/10.1101/2025.09.09.675192; this version posted September 9, 2025. The copyright holder for this preprint (which was not certified by peer review) is the author/funder. This article is a US Government work. It is not subject to copyright under 17 USC 105 and is also made available for use under a CC0 license.

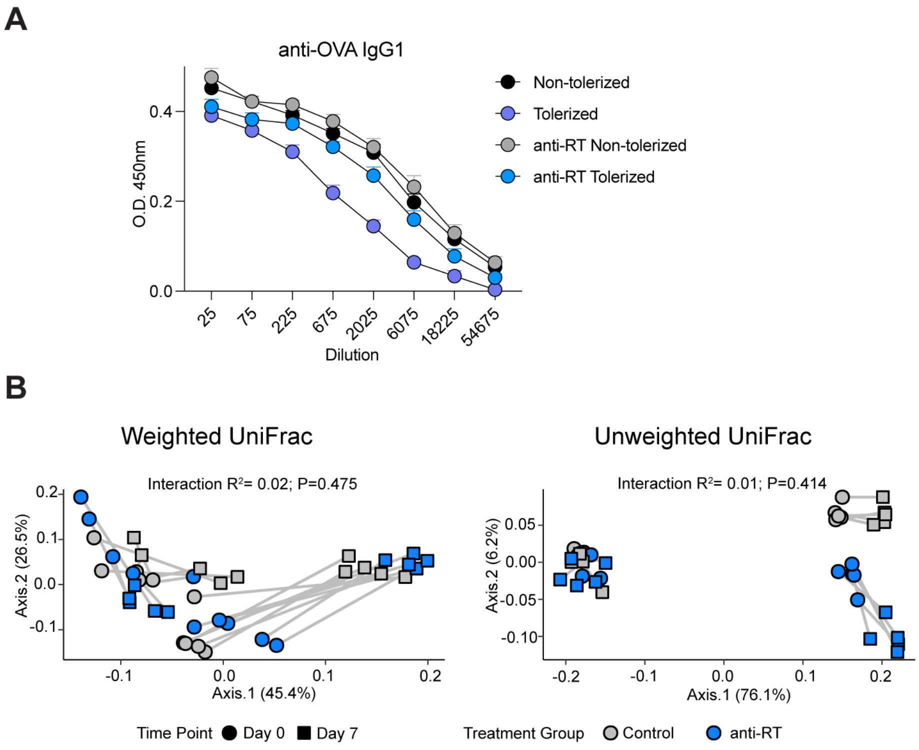

Fig. S1. **Retroelement reverse transcription effect on antibody production and microbiota composition.** (**A**) OVA-specific IgG1 antibodies analyzed by ELISA in sera samples obtained 48h post ear challenge in the DTH model. Graph depicts 1 independent experiment representative of 3 independent experiments. (**B**) Paired analysis of unweighted and weighted UniFrac microbiota 16S profiles in control and anti-RT treated mice, before and after treatment. Percentages represent the variance explained by each PC.

bioRxiv preprint doi: https://doi.org/10.1101/2025.09.09.675192; this version posted September 9, 2025. The copyright holder for this preprint (which was not certified by peer review) is the author/funder. This article is a US Government work. It is not subject to copyright under 17 USC 105 and is also made available for use under a CC0 license.

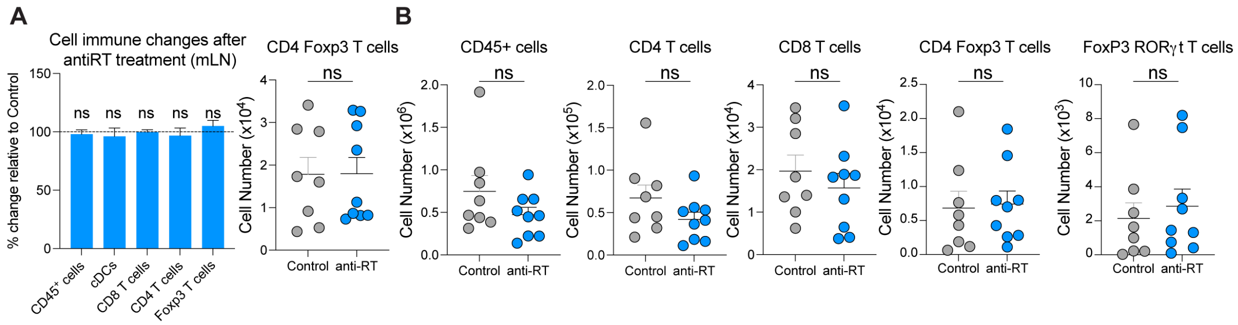

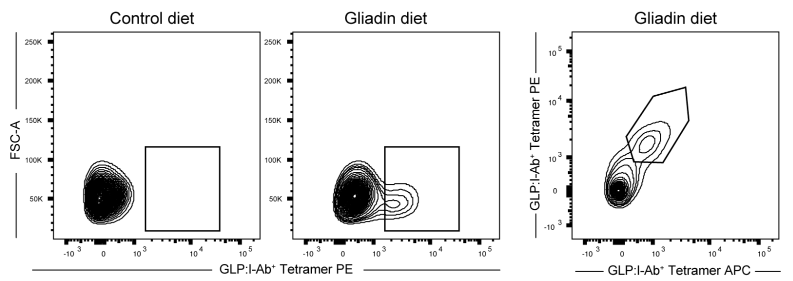

	Control	anti-RT
n1	1.345609	0.96463
	0.516183	0.341246
	1.123418	0.689364
	0.695859	0.288184
	0.826446	0.44953
n2	4.909306	3.969218
	2.347596	3.618421
	3.272059	2.304415
	3.939158	4.352107
	3.827751	1.176684
n3	1.839539	0.1527495
	1.006711	0.2895753
	2.506964	1.861925
	3.542639	1.512674
	1.217499	2.525968

Gliadin Tetramer⁺ cells (x10²)

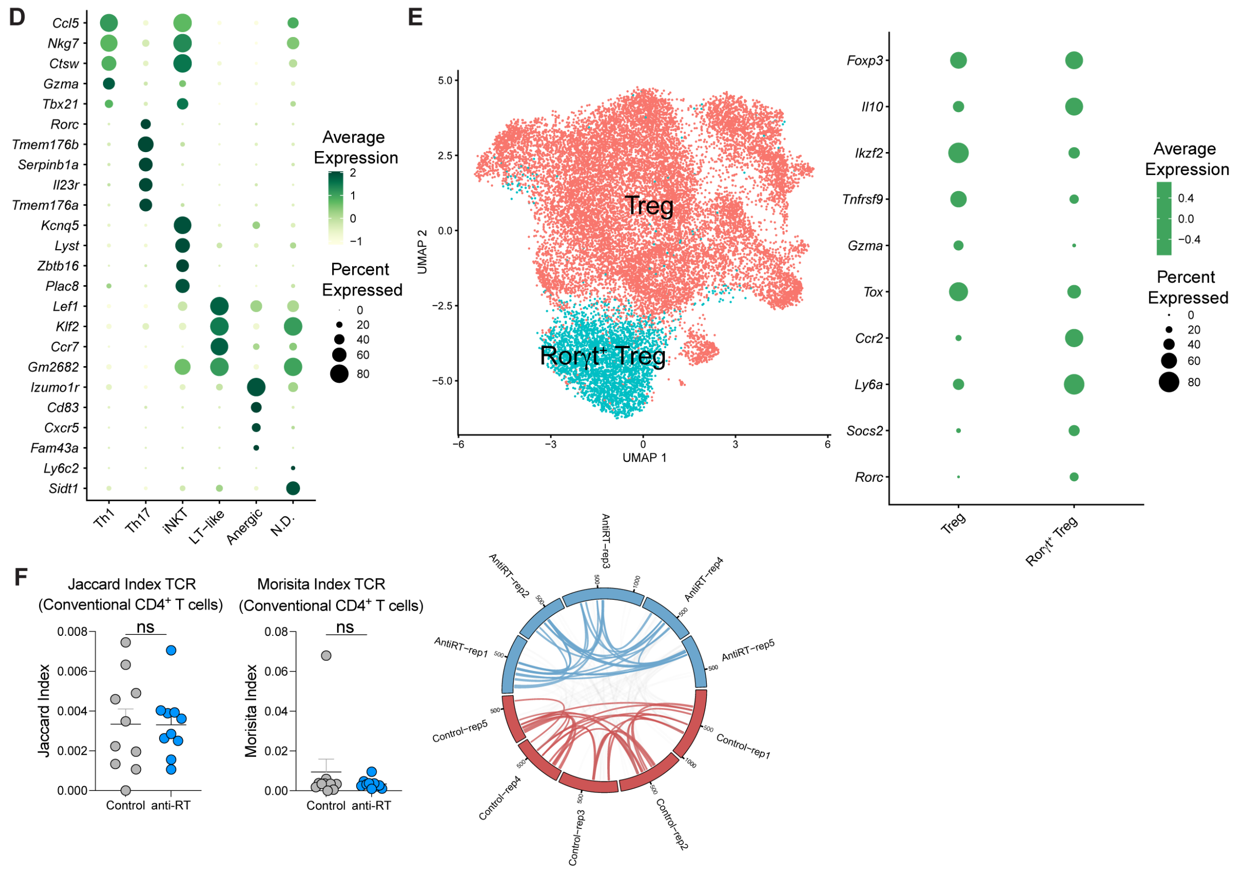

bioRxiv preprint doi: https://doi.org/10.1101/2025.09.09.675192; this version posted September 9, 2025. The copyright holder for this preprint (which was not certified by peer review) is the author/funder. This article is a US Government work. It is not subject to copyright under 17 USC 105 and is also made available for use under a CC0 license.

Fig. S2. Inhibition of retroelement reverse transcription does not influence immune populations in mLN or colon, or TCR clonality in conventional CD4$^+$ T cells. (**A**) (Left) Percentage change relative to control in absolute numbers of the indicated immune populations in mesenteric lymph nodes after 7 days of antiretroviral treatment (left). Live CD45$^+$ CD90.2$^+$ TCRβ$^+$ CD4$^+$ Foxp3$^+$ cells absolute number quantification by flow cytometry analysis in mesenteric lymph nodes (right). (**B**) Live CD45$^+$, Live CD45$^+$ CD90.2$^+$ TCRβ$^+$ CD4$^+$, Live CD45$^+$ CD90.2$^+$ TCRβ$^+$ CD8$^+$, Live CD45$^+$ CD90.2$^+$ TCRβ$^+$ CD4$^+$ Foxp3$^+$ and Live CD45$^+$ CD90.2$^+$ TCRβ$^+$ CD4$^+$ Foxp3$^+$ RorγT$^+$ cells absolute number quantification by flow cytometry analysis in colon. (**C**) Representative contour plot of Glp:I-Ab tetramer$^+$ CD4$^+$ CD44$^+$ Foxp3$^+$ cells in secondary lymphoid organs (SLOs: mLNs, Peyer's patches, spleen and hepatic LNs) and quantification analyzed by flow cytometry. (**D**) Dot plot of highly expressed genes in each cluster from CD4$^+$ T cell single-cell RNAseq analysis, used for cluster annotation. (**E**) UMAP representation of Foxp3$^+$ CD4$^+$ cell clusters from single-cell RNAseq analysis (left). Dot plot of highly expressed genes in each cluster from Foxp3$^+$ CD4$^+$ T cells single-cell RNAseq analysis, used for cluster annotation. (**F**) Jaccard and Morisita indexes of conventional CD4$^+$ T cell TCRs where each dot represents TCR overlap between mice under the same treatment (Left). Circos plot of conventional CD4$^+$ T cell Receptor (TCR) analysis comparing control vs anti-reverse transcriptase treated mice. Each segment represents a mouse. Links between segments represent shared TCR between mice and colored links represent shared TCR between mice under the same treatment (Right). For flow cytometry analyses, data are representative of at least two independent experiments. Each dot represents an individual mouse. Numbers in flow plots indicate mean ± SEM. For (A), (B) and (F) two-tailed unpaired Student's t-test or Mann-Whitney test were used; ns, not significant.

bioRxiv preprint doi: https://doi.org/10.1101/2025.09.09.675192; this version posted September 9, 2025. The copyright holder for this preprint (which was not certified by peer review) is the author/funder. This article is a US Government work. It is not subject to copyright under 17 USC 105 and is also made available for use under a CC0 license.

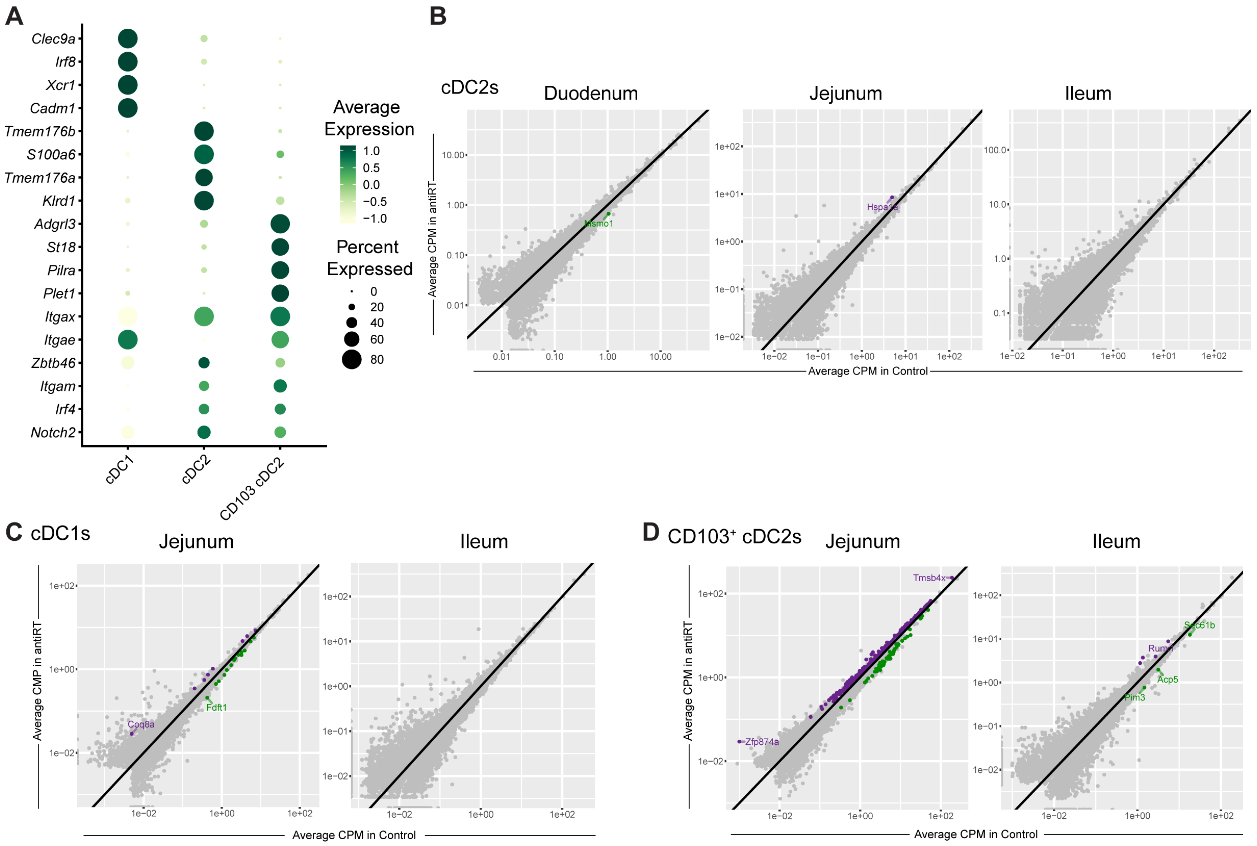

Fig. S3. **Retroelement activity does not modulate dendritic cell populations in the lower parts of the small intestine.** (**A**) Dot plot of highly expressed genes in each cluster from MHC-II^{high} CDC11c^{high} cells single-cell RNAseq analysis, used for cluster annotation. (**B**) Scatter plots of DEGs in cDC2s from duodenum, jejunum and ileum. Purple denotes genes upregulated and green downregulated by anti-RT treatment. (**C**) Scatter plots of DEGs in cDC1s from jejunum and ileum after inhibition of reverse transcriptase treatment. Purple denotes genes upregulated and green downregulated by anti-RT treatment. (**D**) Scatter plots of DEGs in CD103^{+} cDC2s from jejunum and ileum after inhibition of reverse transcriptase treatment. Purple denotes genes upregulated and green downregulated by anti-RT treatment.

bioRxiv preprint doi: https://doi.org/10.1101/2025.09.09.675192; this version posted September 9, 2025. The copyright holder for this preprint (which was not certified by peer review) is the author/funder. This article is a US Government work. It is not subject to copyright under 17 USC 105 and is also made available for use under a CC0 license.

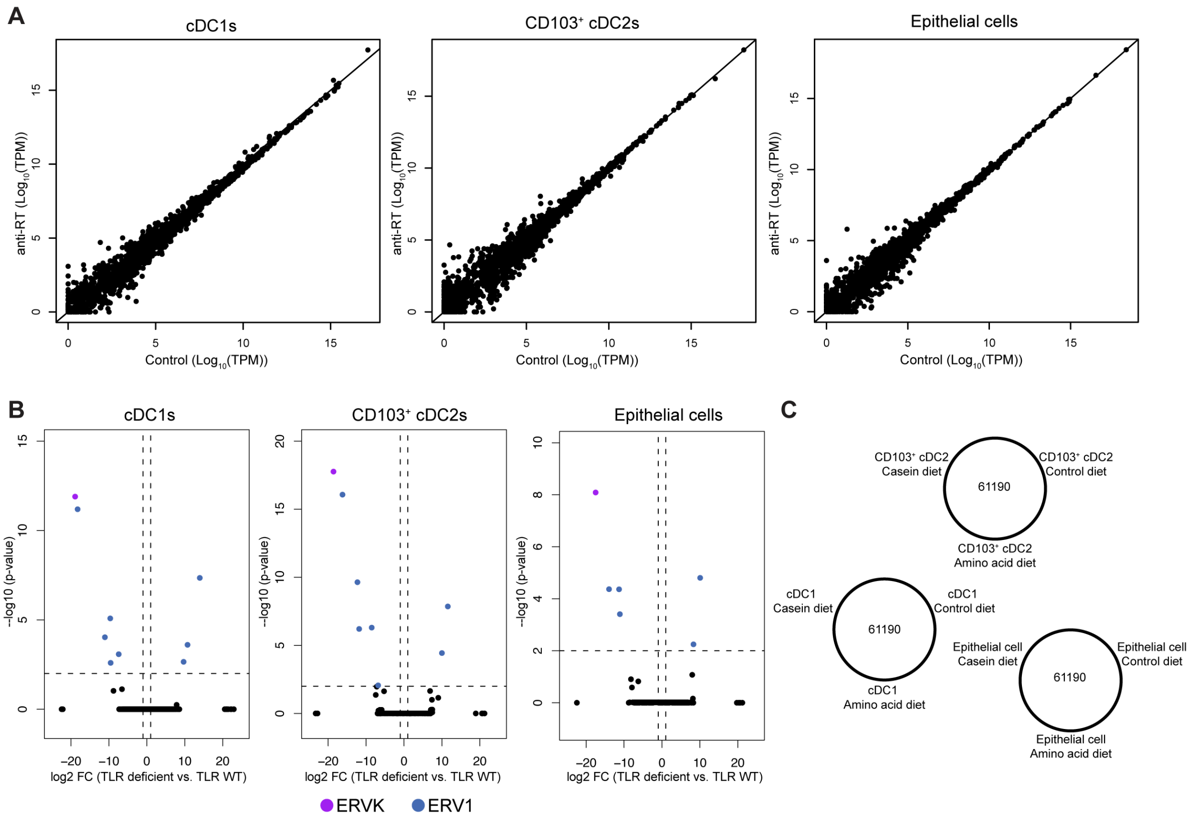

Fig. S4. **Endogenous retroelement expression in small intestine is independent of exogenous factors.** (**A**) Retroelement expression analyzed by bulk RNA sequencing in cDC1s, CD103⁺ cDC2s and epithelial cells from duodenum, in control versus anti-RT treated mice. (**B**) Volcano plots of expressed retroelement loci from cDC1s (left), CD103⁺ cDC2s (middle) and epithelial cells (right) purified from the duodenum of WT or TLR deficient mice. (**C**) Venn diagram of expressed retroelements from bulk RNA-seq analysis. Mice were fed chow, casein, or amino acid (AA) diets since birth, and ERE expression was analyzed in gut epithelial cells, CD103⁺ cDC2s, and cDC1s from duodenum at 8 weeks old.

bioRxiv preprint doi: https://doi.org/10.1101/2025.09.09.675192; this version posted September 9, 2025. The copyright holder for this preprint (which was not certified by peer review) is the author/funder. This article is a US Government work. It is not subject to copyright under 17 USC 105 and is also made available for use under a CC0 license.

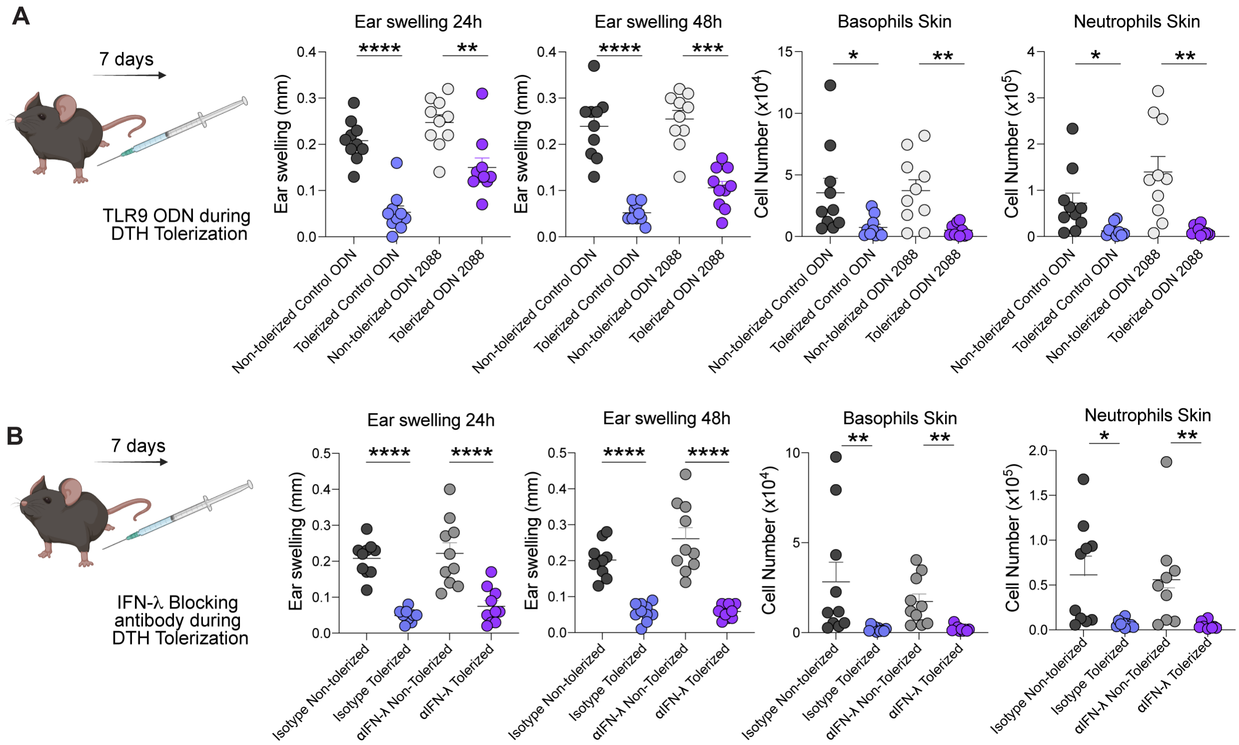

Fig. S5. **TLR9 and IFN-λ are not required for the establishment of oral tolerance.** (**A**) TLR9 signaling pathway was temporally blocked during DTH tolerization using a specific inhibitory oligonucleotide (ODN 2088) or Control ODN. Ear swelling was analyzed after 24h and 48h, and infiltration of neutrophils and basophils were analyzed 48h later by flow cytometry in the skin. (**B**) Mice were treated with a blocking antibody for IFN lambda (2/3) or isotype control during DTH tolerization. Ear swelling was analyzed after 24h and 48h, and infiltration of neutrophils and basophils were analyzed 48h later by flow cytometry. Data are representative of at least two independent experiments. Each dot represents an individual mouse. For (A) and (B), one-way ANOVA with Tukey's multiple comparisons test or Kruskal-Wallis with Dunn's multiple comparisons test were used. Numbers in flow plots indicate mean ± SEM. * $p < 0.05$; ** $p < 0.01$; *** $p < 0.001$; **** $p < 0.0001$.
